# Development of a sensitive plasma lenvatinib quantification method and validation in thyroid cancer patients

**DOI:** 10.1210/jendso/bvag154

**Published:** 2026-07-06

**Authors:** Carla Colombo, Roberta Ottria, Matteo Trevisan, Erika Carbone, Claudia Moneta, Marina Lugaresi, Sara Casati, Sofia Vanerio, Daniele Ceruti, Massimiliano Succi, Valentina Cirello, Simone De Leo, Luca Persani, Pierangela Ciuffreda, Laura Fugazzola

**Affiliations:** Department of Endocrine and Metabolic Diseases, IRCCS Istituto Auxologico Italiano, 20145 Milan, Italy; Department of Pathophysiology and Transplantation, University of Milan, 20122 Milan, Italy; Department of Biomedical and Clinical Sciences, University of Milan, 20157 Milan, Italy; Department of Endocrinology, ASST Santi Paolo e Carlo, 20142 Milan, Italy; Department of Health Sciences, University of Milan, 20142 Milan, Italy; Department of Brain and Behavioral Sciences, University of Pavia, 27100 Pavia, Italy; Department of Health Sciences, University of Milan, 20142 Milan, Italy; Department of Health Sciences, University of Milan, 20142 Milan, Italy; Department of Biomedical, Surgical and Dental Sciences, University of Milan, 20122 Milan, Italy; Fondazione IRCCS Cà Granda, Ospedale Maggiore Policlinico, 20122 Milan, Italy; Department of Biomedical, Surgical and Dental Sciences, University of Milan, 20122 Milan, Italy; Department of Medical Biotechnologies and Translational Medicine, University of Milan, 20129 Milan, Italy; Department of Endocrinology, ASST Santi Paolo e Carlo, 20142 Milan, Italy; Department of Endocrine and Metabolic Diseases, IRCCS Istituto Auxologico Italiano, 20145 Milan, Italy; Department of Pathophysiology and Transplantation, University of Milan, 20122 Milan, Italy; Department of Endocrinology, ASST Santi Paolo e Carlo, 20142 Milan, Italy; Department of Endocrine and Metabolic Diseases, IRCCS Istituto Auxologico Italiano, 20145 Milan, Italy; Department of Medical Biotechnologies and Translational Medicine, University of Milan, 20129 Milan, Italy; Department of Biomedical and Clinical Sciences, University of Milan, 20157 Milan, Italy; Department of Endocrinology, ASST Santi Paolo e Carlo, 20142 Milan, Italy; Department of Health Sciences, University of Milan, 20142 Milan, Italy

**Keywords:** lenvatinib, RAI-refractory, thyroid cancer, plasma quantification, HPLC–MS/MS, adverse events

## Abstract

**Context:**

Lenvatinib (LEN) is effective in the treatment of radioactive iodine refractory differentiated thyroid cancer (DTC), though it is associated with frequent and potentially severe adverse events (AEs). The dose dependency of LEN-related toxicity remains unclear, since no data are available on this topic.

**Objective:**

This study aimed to investigate the relationship between LEN dose and plasma concentrations in real-life patients by a novel and validated method for quantifying LEN in plasma samples.

**Design and setting:**

A sensitive high-performance liquid chromatography coupled with tandem mass spectrometry (HPLC–MS/MS) assay for LEN quantification in plasma was set up, with a wide analytical range (0.7-500 ng/mL). Consecutive plasma samples from 19 patients with radioactive iodine-refractory, metastatic DTC were analyzed.

**Results:**

A positive correlation between the administered LEN dose and plasma concentrations was found, particularly in patients on low doses (<18 mg daily). Linear mixed-effects models (LMMs) demonstrated significant interindividual variability in plasma LEN concentrations, contributing to 17% to 28% of the total variance. This variability was more pronounced at higher doses (≥18 mg), being more likely the consequence of LEN-related AEs than of a dose-dependent effect.

**Conclusion:**

This is the first real-life evaluation of interindividual LEN pharmacokinetics variability in advanced DTC patients, obtained by means of the newly developed HPLC–MS/MS method. Our results suggest that the dose–response relationship of LEN in radioactive iodine-refractory DTC may be more complex than previously understood, with substantial interpatient variability and dose-dependent effects. Further studies are needed to determine the implications of our findings in the clinical management of these patients.

Differentiated thyroid cancer (DTC) has a good prognosis following first-line treatment, which includes surgery and radioactive iodine (RAI) in subjects with a high risk of recurrence [1]. Nevertheless, advanced DTCs may become RAI-refractory, markedly worsening the prognosis, with a median survival of 3-5 years [2]. The development of systemic, lifelong therapy based on multi-tyrosine kinase inhibitors and target drugs has significantly improved outcomes in RAI-refractory DTC. To date, lenvatinib (LEN) is the most widely used multi-kinase inhibitor for the treatment of advanced DTC. LEN efficacy has been extensively demonstrated both in clinical trials [3], and in real-life settings [4-11]. Despite its efficacy, LEN is associated with adverse events (AEs), virtually occurring in all treated patients [3-11], often needing dose reductions (up to 65% of patients) or temporary discontinuation (up to 80%) [3]. These strategies, however, lead to poorer disease control [12, 13], underlying the importance of managing LEN-related toxicities to optimize clinical outcomes. Several key aspects of this management remain unresolved. Indeed, although dose reduction is commonly implemented to mitigate AEs, the existence of a true dose–toxicity relationship is still debated. One phase II trial has suggested that LEN-related AEs may be independent of dosage [12]. Conversely, 2 recent retrospective studies demonstrated that starting LEN treatment at a reduced dose leads to less frequent toxicities [14, 15]. This uncertainty is further reinforced by substantial interindividual variability in LEN pharmacokinetics [16-20], limiting the predictive value of the administered dose for actual drug exposure. Hepatic function [21, 22], sex [21], body weight [23], and genetic polymorphisms affecting LEN absorption and metabolism [24-27] have been proposed as potential causes of this variability. Nevertheless, current evidence remains limited, especially in DTC patients.

This study aimed to investigate for the first time the interindividual LEN pharmacokinetics variability in RAI-refractory DTC patients by means of a newly developed, reliable, and cost-effective method for quantifying LEN plasma concentrations in human samples.

## Materials and methods

### Measurement of LEN concentration in plasma

According to previously validated methods [28-32], the assessment of plasmatic LEN concentration was performed by high-performance liquid chromatography coupled with tandem mass spectrometry (HPLC–MS/MS). Both LEN and the internal standard (IS) LEN-*d*_5_ were obtained by LGC Standards S.r.L. Stock solutions of LEN and LEN-*d*_5_ were prepared in methanol at the concentration of 1 mg/mL and then diluted to obtain working stock solutions at different concentrations (0.1, 1, and 10 μg/mL for LEN, and 0.1 μg/mL for IS).

Samples to build calibration curves (ie, calibration standards, CS) were prepared in pooled blank plasma sampled from healthy volunteers. Different volumes of LEN working solutions were added to obtain decreasing concentrations of 500, 250, 100, 50, 25, 10, and 5 ng/mL. Samples for quality control (QC) were prepared with the same procedure, reaching final concentrations of 250, 25, and 5 ng/mL. Both CS and QC samples, as well as plasma samples from real-life patients treated with LEN, were spiked with the LEN-*d*_5_ working solution to reach a final LEN-*d*_5_ concentration of 20 ng/mL.

All samples (CS, QC, and patients’ samples) were processed using liquid–liquid extraction. Accordingly, 150 µL of cooled acetonitrile (ACN) was added to 50 μL of the sample spiked with the IS. After centrifugation (20 000 g for 10 minutes), extraction was performed with 1-chlorobutane 1 mL. After 10 minutes of automated mixing, samples were centrifuged at 4000 g for 10 minutes. The remnant from organic phase evaporation was diluted in 50 μL of ACNNC/H_2_O (1:1; v/v) containing 0.2% of ammonium acetate.

The HPLC–MS/MS analysis was performed on a 1290 Infinity ultra-high-performance liquid chromatography system coupled to a Q Trap 5500 (ABSciex S.r.l., Milan, Italy), a linear ion trap triple-quadrupole mass spectrometer operating in multiple reaction monitoring mode. The system was equipped with an electrospray ionization source. Chromatographic separation was carried out on a Luna Omega Polar-C18 column (100 mm length × 2.1 mm i.d., 1.6 µm particle size) working at 25 °C, using a linear gradient elution with 2 solvents: 0.1% formic acid (solvent A) and methanol (solvent B). Solvents A and B were 95% and 5% at 0.00 minutes, respectively. Solvent B was increased to 99% from 1.00 to 6.00 minutes, held at 99% from 6.00 to 8.00 minutes, decreased back to 1% from 8.00 to 8.10 minutes, and finally held at 5% from 8.00 to 10.00 minutes for re-equilibration. The flow rate was kept constant at 0.5 mL/min during the analysis. The analyte was ionized by positive electrospray ionization and detected using precursor ions at *m*/*z* 432 and 427 for LEN-*d*_5_ and LEN, respectively, and product ion 370 for both molecules with a collision energy of 39 eV. The ion source worked at a temperature of 500 °C, with an ion spray voltage of 5500 V and a declustering potential of 100 eV. Nitrogen was used as desolvation gas at a pressure of 40 psi. Multiquant software v1.2.1 (ABSciex S.r.l., Milan, Italy) was used for quantification analysis.

### Validation of the method

Prior to applying the method to real-life clinical samples, the method was validated according to the Food and Drug Administration guidelines [33]. Specifically, we assessed linearity, precision, accuracy, sensitivity, specificity, recovery, and matrix effect of the extraction and quantification procedures.

#### Linearity, precision, and accuracy

Calibration curves were generated by spiking QC samples with the IS working solution, as previously described. For each QC sample, the peak area ratio (ie, peak area of LEN/peak area of the LEN-*d*_5_) was plotted against the corresponding nominal concentration, and linear regression was performed. The analysis was repeated 6 times per day for each QC sample, for 3 consecutive days. Intra- and interday accuracy and precision were evaluated on all curve points.

Precision was calculated using equation 1 and is expressed as the coefficient of variation (%CV). Accuracy was calculated using equation 2, where nominal means theoretical amounts, and is denoted by a percentage relative standard error (%RSE).


(1) $$\text{\%}\;\mathrm{CV}=\left(\frac{\mathrm{Standard}\;\mathrm{deviation}}{\mathrm{Mean}\;}\right)\times 100$$

(2) $$\text{\%}\;\mathrm{RSE}=\left(\frac{\mathrm{Standard}\;\mathrm{error}\;\mathrm{nominal}}{\mathrm{Mean}\;}\right)\times 100$$

#### Sensitivity and selectivity

Sensitivity was expressed in terms of limit of detection (LOD) and limit of quantification (LOQ). They were calculated on calibration curves prepared in plasma. The LOD and LOQ were determined by calculating 3.3× and 10× the ratio of the standard deviation (SD) of the response to the slope of the calibration curve, respectively. Stock solutions at concentrations corresponding to the LOD and LOQ were prepared from blank plasma samples and processed in triplicate across 3 consecutive days. To assess selectivity, blank plasma samples were processed in triplicate and analyzed.

#### Recovery

Recovery was evaluated by comparing the peak area ratio of 2 sets of samples. The first set consisted of 3 QC samples (test samples) prepared as described and subjected to the extraction process. For the second set (reference samples), 3 blank plasma samples underwent liquid–liquid extraction, after which 3 aliquots of the supernatant were spiked with the working solutions to match the concentrations of the QC samples (5, 25, and 250 ng/mL for LEN, and 20 ng/mL for LEN-*d*_5_). All samples were analyzed in triplicate. Recovery was defined as the ratio of the peak area of the test sample to the reference sample, expressed as a percentage.

#### Matrix effect

To evaluate matrix effects, QC samples were prepared by spiking blank plasma and methanol samples, after liquid–liquid extraction, with the same concentrations of LEN. Three replicates of each sample were processed. The matrix effect was determined by calculating the percent variation in the peak area ratio of plasma QC samples compared to that of methanol QC samples at the same concentration.

### Application of the method to real-life patients

We retrospectively analyzed 38 consecutive patients with RAI-refractory, progressive metastatic DTC who were treated with LEN at our referral center. The study was performed according to the ethical standards of the Institutional Research Committee and to the Declaration of Helsinki. All patients were enrolled in a protocol approved by the ethical committee of the IRCCS Istituto Auxologico Italiano and provided informed consent to the use of their anonymized clinical data and biological samples for research purposes (study code approval: 2022_03_08_03).

Inclusion criteria were: (1) availability of at least 2 plasma samples collected after LEN reached steady state (ie, after a minimum of 7 consecutive days of treatment); (2) plasma sampling performed at 12:00 ± 3 hours following the most recent LEN intake; and (3) availability of complete demographic and clinical data. Patients who did not meet these criteria, those with impaired renal and/or hepatic function, and those taking drugs potentially interfering with LEN pharmacokinetics (eg, CYP3A4/5, P-glycoprotein, and breast cancer resistance protein inhibitors) were excluded from the analysis. Based on these criteria, a cohort of 19 patients was selected, resulting in 84 plasma samples. Of the 19 excluded patients, 8 had already died before plasma sample collection was initiated, while 2 patients had already switched from LEN to another systemic treatment. The remaining patients who were excluded from the study underwent blood sampling at an external laboratory, which prevented the collection of plasma samples.

LEN was administered orally at a starting dose ranging from 4 to 24 mg daily. LEN starting dose was decided considering the tumor burden, the site of metastatic lesions and the related risk of fistulization, and the patient’s clinical characteristics (age, comorbidities, and performance status). During the follow-up, the LEN dose was either increased, in case of unsatisfactory tumor response or progression, or decreased, in the presence of high-grade or intolerable toxicities. AEs were assessed according to the Common Terminology Criteria for Adverse Events (CTCAE) v5.0.

For each patient, we collected clinical and demographic data both at the start of LEN treatment (baseline) and at each time point of plasma sampling. For this analysis, we included data on sex, body weight, age, and LEN dose at the time of sampling. Regarding LEN-related AEs, data were collected on diarrhea, nausea, vomiting, weight loss, and hypertension.

Blood samples for LEN quantification were collected in 2.7 mL sodium heparin tubes, which were immediately centrifuged at 2500 g for 12 minutes at 4 °C. Plasma was stored at −80 °C until analysis. LEN concentrations were determined using our validated HPLC–MS/MS method.

### Statistical analysis

Categorical variables are expressed as an absolute number and a percentage. For continuous parameters, normality of distribution has been assessed by means of the Shapiro–Wilk test. Continuous variables are described as median and interquartile range (IQR). For calibration curves, the association between the LEN concentration of CSs and the plasmatic concentrations measured by the HPLC–MS/MS assay was assessed using regression analysis with a linear model (*y* = *a* + *bx*).

Using real-life plasma samples, we conducted a linear mixed-effects model (LMM) analysis to evaluate the relationship between administered LEN dose and the corresponding plasma concentrations (plasma LEN). To investigate the relationship between the administered LEN dose and plasma LEN concentrations under various clinical and methodological conditions, several LMMs were fitted (Table 1). Model 1 included all enrolled patients and all collected plasma samples. The study cohort was then stratified according to the administered nominal dose of LEN: Model 2 included patients taking a low LEN dose (<18 mg/day) and the corresponding plasma samples, whereas Model 3 included subjects receiving high doses (≥18 mg/day) and the corresponding plasma samples. Fixed effects were specified for LEN dose in each Model, assuming that a unitary increase of administered LEN dose would produce the same plasmatic LEN increase across patients. Random effects at the patient level were included to account for the correlation among repeated measurements collected over time from the same individual. To investigate the impact of demographic and clinical characteristics on the administered LEN dose and the corresponding plasmatic concentration, we included several covariate adjustments as fixed effects in the LMMs. Given the relatively small sample size compared to the number of included covariates, each model was adjusted for 2 different sets of fixed-effect covariates: SET1 included demographic variables (namely age, sex, and body weight), and SET2 included clinical variables (namely LEN-related AEs). In this context, AEs were treated as a dichotomous factor (present vs absent at any grade, according to CTCAE). For each model, we calculated the intraclass correlation coefficient (ICC) and the slope of the administered LEN dose–plasma LEN concentration curve. The ICC estimated the proportion of total variance attributable to intersubject differences, thus assessing how much of the variability in plasma LEN was due to individual-specific characteristics. The slope estimated the increase of plasma LEN concentration for each mg of orally administered LEN. To compare nested LMMs differing only in their random-effects structure, we used likelihood ratio tests.

**Table 1:** Linear mixed-effects models (LMMs) implemented to evaluate the relationship between administered lenvatinib dose and the corresponding plasma concentrations

Model	Inclusion criteria	No. of patients	No. of plasma samples
1	All available subjects and plasma samples at all time points	19*^a^*	84
2	Plasma samples from patients treated with low LEN doses (<18 mg)	15*^a^*	56
3	Plasma samples from patients treated with high LEN doses (≥18 mg)	8*^a^*	28

Abbreviation: LEN, lenvatinib.

^*a*^During follow-up, lenvatinib doses were both reduced and escalated; thus, plasma samples from the same patient may correspond to either low (<18 mg) or high (≥18 mg) dose levels depending on the sampling time point. Accordingly, some patients contributed samples to both dose groups

The *P*-value for statistical significance has been defined as <.05. All statistical analyses were performed using R (version 4.5.0, R Foundation for Statistical Computing, Vienna, Austria).

## Results

### Method validation

#### Linearity, precision, and accuracy

The calibration curves demonstrated excellent linearity across the entire concentration range (5-500 ng/mL), with a mean coefficient of determination (*R*^2^) of 0.99985 ± 0.0023. Intraday and interday precision and accuracy data are presented in Table S1 [34]. For each QC, the %CV and %RSE were ≤15% and within ±15% of the nominal concentration, respectively.

#### Sensitivity and selectivity

The calculated LOD and LOQ were 0.2 and 0.7 ng/mL, respectively. The analysis of corresponding stock solutions showed values within the acceptable variation limits. Specifically, the signal-to-noise ratio was always greater than 3:1 for the LOD and 10:1 for the LOQ. For the LOQ, the %CV was <20%, and the %RSE was consistently within ±20%.

When blank plasma samples were processed, no significant signals (signal-to-noise ratio >2) from reagents or the biological matrix were detected at the retention time of LEN.

#### Matrix effect and recovery

Matrix effect and recovery results are summarized in Table S2 [34]. Ionization of LEN was slightly increased (6-16%), and this effect was consistent across all evaluated samples, with a %CV ranging from 5.7% to 7.1%. Liquid–liquid extraction allowed a LEN recovery from plasma samples consistently greater than 95%, with variability within the accepted limit of 15% (range 4.3-6.6%).

#### Feasibility of the HPLC–MS/MS assay for routine clinical use

The method requires a small plasma volume (50 µL) and relies on a simple liquid–liquid extraction convenient in terms of time and cost when compared to solid phase extraction (SPE), supporting its suitability for routine clinical sampling. The total chromatographic run time is approximately 10 minutes per sample, including re-equilibration, allowing medium-to-high throughput on standard HPLC–MS/MS platforms and compatibility with laboratories performing therapeutic drug monitoring (TDM). Moreover, the C18 stationary phase used for chromatographic separation is widely employed in hospital laboratories for drug quantification. Implementation requires access to HPLC–MS/MS instrumentation, widely available in hospital and specialized laboratories, and can be readily adopted by trained personnel experienced in bioanalytical methods. Owing to the straightforward sample preparation and lack of expensive consumables, costs are expected to be comparable to other LC–MS/MS-based TDM assays. Overall, the combination of low sample volume, simple workflow, short analysis time, and robust performance supports the feasibility of routine clinical application, potentially enabling individualized LEN dose optimization.

### Application of the method to real-life patients

#### Baseline characteristics of the study cohort

As shown in Table 2, most patients were male (63.2%), with a mean age of 70.4 ± 10.8 years at LEN treatment start. Systemic treatment was started after a mean of 98.2 months from primary tumor diagnosis, with a median starting dose of 20 mg. At the start of LEN treatment, 78.9% of patients had lung metastases, and 36.8% had bone metastases. Other metastatic sites included muscles and subcutaneous tissue in the neck. Patients were followed for a median time of 31 months. Hypertension was the most common toxicity (17/19 patients, 89.5%), being severe (grade ≥3) in most cases (79%). Weight loss was observed in nearly 80% of patients (15/19) and was more commonly moderate (11/15 cases). Nausea and vomiting were less frequent, occurring in 31.6% and 21% of patients, respectively. Severe nausea was reported in only one patient, and no cases of grade ≥3 vomiting were observed. Similarly, no cases of severe diarrhea were recorded among the 13 patients (68.4%) who experienced this side effect.

**Table 2:** Baseline characteristics of the patients enrolled for the analysis of real-life plasma samples

Parameters		Patients, *n* (%)
Sex, F		7 (36.8)
Age at LEN start, years Median (IQR)		68.8 (66.2-77)
Length of disease before LEN start, months Median (IQR)		98.2 (24-164)
ECOG at LEN start	0	14 (73.6)
	1	4 (21.1)
	≥2	1 (5.3)
Primary tumor histotype	Papillary	12 (63.1)
	Follicular	3 (15.8)
	Poorly differentiated	4 (21.1)
T	T1a-b	1 (5.3)
	T2-T3a	5 (26.3)
	T3b	11 (57.9)
	T4a-b	2 (10.5)
N	Nx/N0a-b	8 (42.1)
	N1a-b	11 (57.9)
M	Mx/M0	17 (89.4)
	M1	2 (10.6)
Distant mts at LEN start	Lung	15 (78.9)
	Bone	7 (36.8)
	Others	3 (15.8)
LEN starting dose, mg Median (IQR)		20 (10-20)
Treatment duration, months Median (IQR)		31 (15-38)

Abbreviations: ECOG, Eastern Cooperative Oncology Group; F, female; IQR, interquartile range; LEN, lenvatinib; mts, metastases; SD, standard deviation.

A total of 84 plasma samples were analyzed, with the majority collected from patients receiving a 10 mg dose (30/84, 35.7%).

#### Comprehensive analysis of real-life plasma samples

**Figure 1:**
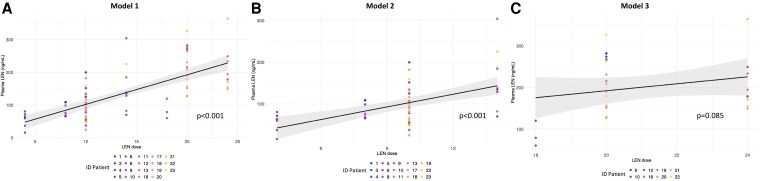
Linear mixed-effects models, before adjusting for covariates, showing the relationship between LEN dose (*x*-axis) and plasma LEN (*y*-axis): (A) Model 1 (plasma samples from all patients); (B) Model 2 (plasma samples from patients treated with LEN <18 mg); (C) Model 3 (plasma samples from patients treated with LEN ≥18). During follow-up, lenvatinib doses were both reduced and escalated; thus, plasma samples from the same patient may correspond to either low (<18 mg) or high (≥18 mg) dose levels depending on the sampling time point. Accordingly, some patients contributed samples to both dose groups.

The estimated slopes, ICC, and *P*-values for each LMM upon adjustment for covariates SET1 and SET2 are shown in Table 3. In Model 1, the administered LEN dose demonstrated a strong and highly significant positive correlation with plasma LEN (*P* < .001), both before (Fig. 1A) and after adjustment for both SETs of covariates. For each additional mg of LEN administered, plasma LEN increased from 8.48 to 9.70 ng/mL. When adjusting for demographic variables (SET1), the ICC was 0.17, suggesting that about 20% of the total variance in plasma LEN was due to interpatient differences. When adjusting for AEs (SET2), the ICC increased to 0.278, reflecting greater interpatient variability. Given the skewed age distribution of our study cohort toward advanced age, and the potential impact of age on LEN PKs, we conducted univariable LMMs evaluating the association between age and plasma LEN concentrations, in which an apparent association was observed (*data not shown*). Age was subsequently included as a covariate in the final LMMs, together with nominal LEN dose, body weight, and sex. In the multivariable models, age was not found to be a statistically significant predictor of plasma LEN concentrations (*data not shown*).

**Table 3:** Slope and interclass correlation coefficient estimates, with associated *P*-value, of the relationship between administered lenvatinib dose and the corresponding plasma concentrations, according to each linear mixed-effects model implemented in the analysis

Fixed-effect covariates	Model 1 All patients and all plasma samples		Model 2 Plasma samples from patients taking LEN < 18 mg		Model 3 Plasma samples from patients taking LEN ≥ 18 mg	
	Slope (*P*-value)	ICC	Slope (*P*-value)	ICC	Slope (*P*-value)	ICC
Demographic (sex, age, body weight)	9.70 (<.001)	0.17	8.27 (.019)	0.207	13.58 (.109)	0.524
Clinical (LEN-related AEs)	8.48 (<.001)	0.278	10.66 (.00038)	0.261	4.97 (.533)	0.385

Abbreviations: AEs, adverse events; ECOG, Eastern Cooperative Oncology Group; ICC, intraclass correlation coefficient; LEN, lenvatinib.

#### Analysis of plasma samples upon stratification for administered LEN dose

In the subgroup of patients treated with low LEN doses (<18 mg, Model 2), the correlation between administered dose and corresponding plasmatic level was statistically significant both before (Fig. 1B) and after adjustment for covariates SET1 and SET2 (*P* = .019 and *P* = .00038, respectively). Conversely, in the high dose subgroup (≥18 mg, Model 3), the effect of LEN dose on plasma LEN was not statistically significant either before (Fig. 1C) or after adjustment for covariates SET1 (*P* = .109) and SET2 (*P* = .533).

When adjusting for demographic variables (SET1), interpatient variability was substantially lower in the low-dose group (21%) compared to the high-dose group (52%). Despite a slight reduction, this difference remained relevant even after adjusting for SET2 (26% *vs* 39%). Notably, correction for different SETs of fixed-effect covariates had minimal impact on the ICC within the low-dose subgroup (0.207 for SET1 vs 0.261 for SET2), while a substantial reduction was observed in the high-dose group (0.524 for SET1 vs 0.385 for SET2).

The effect of the administered LEN dose on the interindividual variability was confirmed by dose-specific ICC values obtained by stratifying Model 1 according to the administered LEN dose. The ICC progressively increased with higher doses, ranging from 0.016 at the lowest dose of 4 mg to 0.671 at the highest dose of 24 mg. The most notable increase occurred between the 14 and 18 mg doses, where the ICC increased from 0.298 to 0.48 (Table S3) [34].

#### Impact of administered LEN dose on the corresponding plasma concentration

To investigate the difference of ICC values between patients treated with low and high LEN doses, we reassessed each LMMs including LEN dose at the patient level as a random effect. Thus, we allowed the dose–response relationship to vary across both different individuals and increasing dosages of administered LEN. The results from this analysis (Table 4) were consistent with those from the corresponding models that did not account for the random effect of LEN dose, and no significant differences were registered when comparing the paired models (*P* > .05 in all cases). This suggested that the same increase of administered LEN dose produces a comparable increase across different subjects. We also extracted individual patient-specific random intercepts and slopes from the most comprehensive Model 1, evaluated with both SETs of covariates (Table S4) [34]. The variance (*σ*^2^) analysis revealed greater heterogeneity in the distribution of random intercepts compared to random slopes for both demographic (*σ*^2^_intercept_/*σ*^2^_slope_ = 20.7) and clinical covariates (*σ*^2^_intercept_/*σ*^2^_slope_ = 34.2).

**Table 4:** Slope and interclass correlation coefficient estimates, with associated *P*-value, of the relationship between administered lenvatinib dose and the corresponding plasma concentrations, according to each linear mixed-effects model implemented in the analysis after including a random effect for LEN dose at the patient level

Fixed-effect covariates	Model 1 All patients and all plasma samples		Model 2 Plasma samples from patients taking LEN < 18 mg		Model 3 Plasma samples from patients taking LEN ≥ 18 mg	
	Slope (*P*-value)	ICC	Slope (*P*-value)	ICC	Slope (*P*-value)	ICC
Demographic (sex, age, body weight)	10.4 (<.001)	0.198	7.51 (.0263)	0.242	16.47 (.0771)	0.561
Clinical (LEN-related AEs)	8.35 (<.001)	0.285	9.32 (.00023)	0.295	6.87 (.426)	0.417

Abbreviations: AEs, adverse events; ECOG, Eastern Cooperative Oncology Group; ICC, intraclass correlation coefficient; LEN, lenvatinib.

## Discussion

This is the first real-life evaluation of interindividual LEN pharmacokinetics variability in advanced DTC patients. MKIs therapy is associated with variable toxicities among different patients, which cannot be entirely explained by the administered drug dose, since some patients experience high toxicity at low doses, while others are able to tolerate high doses. Given the interindividual variability in LEN pharmacokinetics, the most probable mechanism to explain what is observed in clinical practice, we set up and validated a method to measure LEN concentration in plasma samples using HPLC–MS/MS. The assay was highly sensitive, allowing the precise assessment of LEN in a range of concentrations (0.7-500 ng/mL) wide enough to cover all real-life therapeutic samples, and the chromatographic separation provided optimal specificity. Consequently, we obtained clean chromatograms with no co-eluting substances at the retention time of LEN (3.27 minutes). All other validation parameters related to extraction and quantification met FDA criteria. The method requires a small plasma volume (50 µL) and a simple workflow, a short analysis time, and a robust performance supporting the feasibility of routine clinical application, potentially enabling individualized LEN dose optimization.

The application of our HPLC–MS/MS assay to plasma samples from real-life patients revealed a linear correlation between the nominal administered LEN dose and plasma concentrations in the whole group. We expected this finding to be valid for each LEN dose, with the lack of statistical significance in the subgroup of patients receiving higher LEN doses (18-24 mg) possibly explained by the small number of plasma samples (*n* = 28) and by the narrow dose range in this subgroup. This may have led to overlapping plasma LEN concentrations, further reducing the detectability of a significant dose–response relationship. The age distribution of the study cohort was shifted toward older individuals. Notably, the multivariable LMMs revealed that age does not independently contribute to the variability in LEN exposure after accounting for relevant clinical covariates, consistent with previous data [16], and indicating that our findings are also applicable to younger patients.

The ICC values derived from our most comprehensive LMM (Model 1) confirmed substantial interindividual variability in plasma LEN concentrations, contributing to 17% to 28% of the total variance, which aligns with previous reports on both healthy subjects and patients with solid tumors [16-20]. Particularly, a prior population pharmacokinetics study including clinical trials on DTC patients reported coefficients of variation of 25% for LEN clearance and 38% for exposure, as measured by the area under the curve [16]. Notably, in our study, ICC values varied significantly when plasma samples were stratified by LEN dose, with higher values observed in the high-dose subgroup (Model 3) compared to the low-dose subgroup (Model 2). Accordingly, we hypothesized that the slope of the LEN dose–plasma LEN relationship could differ among patients. However, including LEN dose as a random effect to account for individual-specific slopes did not significantly improve the models’ performance, implying that increments in LEN dose produce similar plasma concentration changes across individuals. This was further supported by the observation that random intercepts exhibited greater variance than random slopes, indicating that patients receiving the same LEN dose show consistently different plasma concentrations at steady state. This variability is likely due to several factors, such as different plasma albumin levels (which bind more than 98% of circulating LEN and can be significantly altered during LEN treatment) [22], genetic differences in LEN metabolism (CYP3A4/CYP3A5 polymorphisms) [27], and transporter-mediated absorption/excretion (P-glycoprotein and breast cancer resistance protein inhibitors, encoded by ABCB1 and ABCG2, respectively) [24-26, 35, 36]. Particularly, a real-world Japanese study involving 40 advanced DTC patients demonstrated that steady-state plasma LEN concentrations varied significantly according to CYP3A4 polymorphisms, with a similar trend observed for CYP3A5 and ABCG2 [27]. Similarly, Cantara et al found that polymorphisms in CYP3A4/5, ABCB1, and ABCG2 genes were significantly associated with different frequencies and severities of LEN-related toxicities, though LEN exposure was not evaluated [37]. In our study, the ICC values for Model 2 remained stable regardless of the covariates included, whereas those for Model 3 decreased markedly (from 0.524 with demographic covariates to 0.385 with clinical covariates). These findings suggest a potential interaction between LEN dose and associated toxicities, with higher LEN doses potentially causing greater heterogeneity in AEs. Our models seem to indicate that LEN-related toxicities, particularly vomiting and diarrhea, could impair absorption and contribute to increased interpatient variability at higher doses, and suggest reconsidering the current recommendation to administer a fixed 24 mg dose of LEN for all RAI-refractory DTC patients [38].

These results, obtained in the first series of thyroid cancer patients on LEN, are promising and consistent with what is expected from the real-life clinical practice. However, they are preliminary and subjected to the limitation to be retrospective and obtained in a relatively small sample, thus limiting the complexity of the models, reducing statistical power, and potentially affecting the generalizability of the findings. Moreover, only a limited number of plasma samples were collected per patient (3-7 samples), and samples for each subject were obtained under no more than 2 distinct LEN doses. Consequently, intraindividual variability and its temporal evolution could not be assessed. Similarly, the wide spacing of plasma sampling time points prevented a reliable estimation of cumulative LEN exposure over time, precluding the assessment of a potential correlation between LEN exposure and tumor response to treatment, as well as the identification of a plasma LEN cutoff associated with a better outcome.

In conclusion, we developed and validated a reliable HPLC–MS/MS method for quantifying LEN concentrations in plasma. This assay was successfully applied to plasma samples from real-life patients, revealing a linear correlation between the nominal administered LEN dose and plasma concentrations and a consistent interindividual variability in LEN metabolism, likely due to both inherent biological factors and dose-dependent effects. Further studies are needed to determine the clinical implications of these findings in advanced thyroid cancer patients on LEN. Hopefully, the precise quantification of LEN exposure may allow the identification of factors significantly influencing LEN PK. In turn, this could pave the way for a patient-tailored approach based on LEN dose individualization, maximizing treatment response while minimizing treatment-related toxicities.

## Data Availability

The data that support the findings of this study are available on request from the corresponding author.
